# Improving the use of research evidence in guideline development: 6. Determining which outcomes are important

**DOI:** 10.1186/1478-4505-4-18

**Published:** 2006-12-01

**Authors:** Holger J Schünemann, Andrew D Oxman, Atle Fretheim

**Affiliations:** 1 INFORMA, S.C. Epidemiologia, Istitituto Regina Elena, Via Elio Chianesi 53, 00144 Rome, Italy; 2 Norwegian Knowledge Centre for the Health Services, P.O. Box 7004, St. Olavs plass, N-0130 Oslo, Norway

## Abstract

**Background:**

The World Health Organization (WHO), like many other organisations around the world, has recognised the need to use more rigorous processes to ensure that health care recommendations are informed by the best available research evidence. This is the sixth of a series of 16 reviews that have been prepared as background for advice from the WHO Advisory Committee on Health Research to WHO on how to achieve this.

**Objectives:**

We reviewed the literature on determining which outcomes are important for the development of guidelines.

**Methods:**

We searched five databases of methodological studies for existing systematic reviews and relevant methodological research. We did not conduct a complete systematic review ourselves. Our conclusions are based on the available evidence, consideration of what WHO and other organisations are doing and logical arguments.

**Key questions and answers:**

We did not find a systematic review that addresses any of the following key questions and we found limited relevant research evidence.

What methods should WHO use to identify important outcomes?

• Methods of outcome identification should be transparent and explicit.

• The consultation process should start with identification of all relevant outcomes associated with an intervention.

• Those affected, including consumers, should be involved in the selection of outcomes.

• A question driven approach (what is important?) is preferable to a data driven approach (what data are at hand?) to identify important outcomes.

What type of outcomes should WHO consider and how should cultural diversity be taken account of in the selection of outcomes?

• Desirable (benefits, less burden and savings) and undesirable effects should be considered in all guidelines.

• Undesirable effects include harms (including the possibility of unanticipated adverse effects), greater burden (e.g. having to go to the doctor) and costs (including opportunity costs).

• Important outcomes (e.g. mortality, morbidity, quality of life) should be preferred over surrogate, indirect outcomes (e.g. cholesterol levels, lung function) that may or may not correlate with patient important outcomes.

• Ethical considerations should be part of the evaluation of important outcomes (e.g. impacts on autonomy).

• If the importance of outcomes is likely to vary across cultures, stakeholders from diverse cultures should be consulted and involved in the selection of outcomes.

How should the importance of outcomes be ranked?

• Outcomes should be ranked by relative importance, separated into benefits and downsides.

• Information from research on values and preferences should inform the ranking of outcomes whenever possible.

• If the importance of outcomes is likely to vary across cultures, ranking of outcomes should be done in specific settings.

• If evidence is lacking for an important outcome, this should be acknowledged, rather than ignoring the outcome.

## Background

The World Health Organization (WHO), like many other organisations around the world, has recognised the need to use more rigorous processes to ensure that health care recommendations are informed by the best available research evidence. This is the sixth of a series of 16 reviews that have been prepared as background for advice from the WHO Advisory Committee on Health Research to WHO on how to achieve this.

An outcome can be defined as a measure of an intervention's desirable (benefits, less burden and savings) or undesirable effects (including harms, greater burdens and cost). Those making health care recommendations always should consider the benefits, potential harms, including the potential for unanticipated adverse effects, burdens (e.g. having to take a pill), and costs, including opportunity costs. Identifying all known and plausible outcomes that are important to those affected and associated with an intervention is a key step in formulating questions for guideline development. Unfortunately, guideline developers sometimes select outcomes based on what has been assessed in studies rather than based on what is important to those affected.

Since interventions affect several outcomes (e.g. some hypertensive treatments have effects on mortality, stroke, diabetes, libido), guideline developers need to consider their relative importance. This is also true for public health and health systems interventions. For example, media campaigns might cause anxiety as well as promoting a desired health behaviour, and there are always associated costs. At the very least, there are opportunity costs.

Patients may assign different values to outcomes than clinicians and clinical experts involved in guideline development [1]. In addition, surrogate outcomes such as laboratory measures that are part of the clinician's repertoire often do not correlate with patient important outcomes and guideline developers should scrutinize surrogate outcomes about how directly they relate to patient important outcomes.

In this paper we addressed the following questions:

• What methods should WHO use to identify important outcomes?

• What type of outcomes should WHO consider and how should cultural diversity be taken account of in the selection of outcomes?

• How should the importance of outcomes be ranked?

Questions related to integrating values and consumer involvement are specifically addressed in another paper in this series [2].

## What WHO is doing now

The Guidelines for WHO Guidelines suggests the following:

• "To identify the issues to be addressed, it is helpful to develop a logic and analytical frameworks guide (Woolf, 1994)" [3]. (GWG 6C1 Process of developing guidelines)

• "Spell out any tradeoffs between the cost of applying possible recommendations on a population basis, and the population health impacts" in the second stage of guideline development. (section 5d Making recommendations)

• "All evidence, including that on safety, should be clearly laid out in an evidence table" (GWG section 6C2).

Despite these guidelines, a review of several WHO guidelines (e.g., contraceptive use, hypertension, air pollution, inpatient treatment of malnourished children, treatment of non-breastfed children) revealed that the process of outcome identification is usually not described.

## What other organisations are doing

The UK National Institute for Health and Clinical Excelence (NICE) defines a very explicit process for the identification of outcomes using the Population, Intervention, Comparison and Outcome (PICO) format for the development of questions [4]. The NICE handbook asks guideline panels to consider:

• What outcome is really important for the patient?

• Which outcomes should be considered: intermediate or short-term measures (e.g., mortality, morbidity and treatment complications, quality of life, cost, etc)?

Similarly, the National Health and Medical Research Council of Australia bases its approach on the NICE handbook and defines the appropriateness of the outcomes by asking "Are they relevant to the patient?" [5,6].

SIGN underlines (section 5.1. of the SIGN handbook for guideline developers) that patients' perspectives should be included early in the guideline development process [7]. Therefore, SIGN prescribes to conduct a specific literature search designed to cover both quantitative and qualitative evidence about outcomes without limitations of study design, but this is not (yet) done consistently (Robin Habour, personal communication). In theory, the results of this search inform the development of key questions. SIGN uses the PICO format for question development.

The United States Preventive Services Taskforce (USPSTF) [8] describes that value judgments are involved in using the information in an outcomes table to rate either benefits or harms. USPSTF uses a 4-point scale to rate importance. Value judgments are also needed to weigh benefits against harms and to arrive at a rating of net benefit. The USPSTF does not use formal processes for identifying outcomes as part of the question formulation. Specialty societies do not consistently acknowledge a formal process for question development and the processes are often not transparent.

## Methods

The methods used to prepare this review are described in the introduction to this series [9]. Briefly, the key questions addressed in this paper were vetted amongst the authors and the ACHR Subcommittee on the Use of Research Evidence (SURE). We did not conduct a full systematic review. We reviewed existing guidelines for guidelines to identify processes for outcome identification and ranking. We also searched PubMed using (guideline OR policy making) and (identification) and (outcomes) as search terms (MESH headings/keywords) for systematic reviews and studies of methods for identifying outcomes for guideline development (69 citations). We also searched the Cochrane Methodology Register and Database of Methodology Reviews using the keywords "outcome" and "identification". We also searched databases maintained by the Agency for Healthcare Research and Quality (AHRQ, [10]) and the Guidelines International Network (GIN, [11]). These searches were supplemented with information obtained directly from guideline development organizations and our own files. The answers to the questions are our conclusions based on the available evidence, consideration of what WHO and other organisations are doing and logical arguments.

## Findings

We did not find a systematic review that addresses any of the key questions and we found very little relevant research evidence.

## What methods should WHO use to identify important outcomes?

Few guideline developers have included descriptions of methods for the identification of important outcomes. SIGN uses an approach that begins with conducting a search for evidence using the patient perspective before finalizing the formation of the question. Most other guideline developers have not described formal processes of identifying important outcomes when formulating guideline questions. To be reproducible and understandable, the methods of outcome identification should be transparent and explicit.

NICE suggests facilitating the process of formulating questions, "it may be helpful to construct a diagram listing outcomes and other key criteria the [guideline] group has considered important. Once the question has been framed, key words can be identified as potential search terms" [12]. NICE involves patient organistions in developing guideline scopes and routinely includes at least two patient or caregiver members who provide a patient perspective on all guideline development activities including the formulation of clinical questions and defining of relevant outcomes [13]. Owens and Nease suggest the use of influence diagrams to identify important outcomes and focus guideline questions [14]. They argue this helps to delineate an explicit link between interventions and outcomes, shifts the focus from broad questions to more sharply delineated questions to be addressed, and highlight the importance of a clear, unambiguous statement of whose benefit, downsides and costs are under consideration. Thus, this limited evidence suggests that a consultation process should start with identification of all relevant outcomes associated with an intervention.

Bravata and colleagues. conducted an overview of reviews to identify innovative methods for question formulation related to challenging topics in health care (organization, delivery and financing of health care) [15]. They found that the use of decision analytical frameworks for defining a question and systematic methods such as influence diagrams influenced how questions were formulated. Furthermore, systematic methods appeared to have an effect on search strategies to identify underlying evidence.

There is little empirical evidence to inform decisions about what methods to use to identify important outcomes. Given the paucity of data on patients' and the public's values WHO should consider using other evidence such as systematic summaries and original research on people's perspectives and experiences ("views" studies) alongside trials of effectiveness. Summarizing views studies in a systematic way could lead to a greater breadth of perspectives and a deeper understanding of public health issues from the point of view of those targeted by interventions. Harden et al. suggest that this methodology is likely to create greater opportunities for people's own perspectives and experiences to inform policies to promote their health [16].

Compared to the limited evidence about methods to identify important outcomes in guidelines, there is a large literature that documents that the importance of outcomes can vary within and across cultures, and between health care professionals and patients [1,17]. In addition, both clinical and public health interventions can have effects that are important to consumers, but are not considered important by researchers or health professionals in part because values differ between decision makers. This suggests two key elements of any approach that is used to identify important outcomes. First, all relevant stakeholders (including consumers) should be consulted at an early stage in the process. Secondly, the formulation of questions and the search for evidence should then consider all relevant outcomes.

## What type of outcomes should WHO consider and how should cultural diversity be taken account of in the selection of outcomes?

The AGREE Collaboration states that the guidelines development process "involves taking into account the benefits, harms and costs of the recommendations, as well as the practical issues attached to them" [18]. The AGREE instrument suggests guidelines "should consider health benefits, side effects, and risks of the recommendations. For example, a guideline on the management of breast cancer may include a discussion on the overall effects on various final outcomes. These may include: survival, quality of life, adverse effects, and symptom management or a discussion comparing one treatment option to another. There should be evidence that these issues have been addressed." It also suggests that the potential cost implications of applying the recommendations should have been considered. In general, desirable and undesirable effects should be considered in all guidelines. Undesirable effects include harms (including the possibility of unanticipated adverse effects), burdens (e.g. having to go to the doctor) and costs (including opportunity costs).

The GRADE Working Group suggests that explicit judgements should be made about which outcomes are critical, which ones are important but not critical, and which ones are unimportant and can be ignored. The group emphasizes that all important outcomes should be considered in making a recommendation, but only critical ones should be considered when making judgements about the overall quality of the evidence underlying a recommendation [19]. They recommend that it is important to consider costs (resource utilisation) before making a recommendation. They also suggest that studies using surrogate outcomes generally provide weaker evidence than those using outcomes that are important, and these only should be included when evidence for important outcomes is lacking. Thus, important outcomes (e.g. mortality, morbidity, quality of life) should be preferred over surrogate, indirect outcomes (e.g. cholesterol levels, lung function) that may or may not correlate with patient important outcomes.

Because the importance of different outcomes can vary dramatically and the importance attached to different outcomes may vary from culture to culture, it is important to take cultural diversity into account when deciding which outcomes are important [20-23]. Prenatal screening and genetic counseling are examples of interventions for which the importance of an outcome (abortion) varies between individuals and across cultures, because of religious beliefs or values [24,25]. End of life decisions are influenced by the roles of decision makers (clinician versus patient and family) and cultural differences [20,22]. The choice of using aspirin is related to the values and preferences of diabetic patients and patients place very different values on preventing strokes than their health care providers [1,26]. Cultural differences can be taken into account through the involvement of stakeholders from different cultures, and may require that judgments about trade-offs between the benefits and downsides of an intervention are specific for different cultures [27-29]. Values of stakeholders should be elicited and transparently described in recommendations. We offer strategies in another article of this series [2].

Ethical considerations should also be taken into account when selecting outcomes. For example, with directly observed therapy for tuberculosis, individual rights to refuse therapy (autonomy) may have to be sacrificed for the benefit of society [30]. Explicit identification of ethical consequences, and explicit judgments about trade-offs such as these, can help to ensure that appropriate judgments are made, help to resolve or clarify disagreements, and facilitate local adaptation of guidelines.

## How should the importance of outcomes be ranked?

Judgments about the balance between the benefits and downsides of an intervention require judgments about the relative importance of the different outcomes, either explicitly or implicitly. Ranking outcomes by their relative importance, separated into benefits and downsides in an evidence profile [7,12] can help to focus attention on those outcomes that are considered most important, and help to resolve or clarify disagreements. Research on values and preferences should guide the ranking of outcomes, whenever possible. Guideline panels may want to search for research on the values associated with specific outcomes of interest to inform judgments about their relative importance.

If the importance of outcomes varies across cultures, ranking should be done by people in a specific setting, who can pay due consideration to local values and preferences. If evidence is lacking for an important outcome, this should be acknowledged, rather than ignoring the outcome.

## Discussion

There is very limited evidence to inform decisions about how to select and rank outcomes. However, we recommend the use of systematic and transparent methods involving key stakeholders, including consumers and people from different cultures, to help ensure that all important outcomes are considered and facilitate local adaptation of guidelines. Limitations of our work include the possibility that we have missed relevant studies.

## Further work

Although it is possible that there is relevant empirical research of which we are not aware, a complete systematic review of the questions addressed in this paper is unlikely to change the conclusion that there is very little research evidence in this area. Evaluations comparing different methods of identifying, selecting and ranking outcomes are needed.

## Competing interests

ADO and AF work for the Norwegian Knowledge Centre forthe Health Services, an agency funded by the Norwegian government that produces systematic reviews and health technology assessments. All three authors are contributors to the Cochrane Collaboration. ADO and HJS are members of the GRADE Working Group. HJS is documents editor and chair of the documents development and implementation committee for the American Thoracic Society and senior editor of the American College of Chest Physicians' Antithrombotic and Thrombolytic Therapy Guidelines.

## Authors' contributions

HJS prepared the first draft of this introduction. ADO and AF contributed to drafting and revising it.

## References

[B1] Devereaux PJ, Anderson DR, Gardner MJ, Putnam W, Flowerdew GJ, Brownell BF, Nagpal S, Cox JL (2001). Differences between perspectives of physicians and patients on anticoagulation in patients with atrial fibrillation: observational study. Bmj.

[B2] Schünemann HJ (2006). Improving the Use of Research Evidence in Guideline Development: 10. Integrating values and consumer involvement. Health Res Policy Syst.

[B3] Woolf SH, DiGuiseppi CG, Atkins D, Kamerow DB (1996). Developing evidence-based clinical practice guidelines: lessons learned by the US Preventive Services Task Force. Annu Rev Public Health.

[B4] McKibbon A, Hunt D, Richardson SW, al., Guyatt G RD (2002). Finding the evidence. Users' Guides to the Medical Literature: A Manual for Evidence-Based Clinical Practice.

[B5] National Health and Medical Research C (2000). Handbook series on preparing clinical practice guidelines. February 2000. Australia..

[B6] National Health and Medical Research C NHMRC additional levels of evidence and grades for recommendations for developers of guidelines PILOT PROGRAM 2005.

[B7] Scottish Intercollegiate Guideline Network. SIGN 50: A guideline developers' handbook. Section 5.. http://www.sign.ac.uk/guidelines/fulltext/50/section5.html.

[B8] Harris RP, Helfand M, Woolf SH, Lohr KN, Mulrow CD, Teutsch SM, Atkins D (2001). Current methods of the U.S. Preventive Services Task Force:  A review of the process. American Journal of Preventive Medicine.

[B9] Oxman AD (2006). Improving the Use of Research Evidence in Guideline Development: Introduction. Health Res Policy Syst.

[B10] Agency for Healthcare Research and Quality. http://www.guidelines.gov.

[B11] Guidelines International Network. http://www.g-i-n.net.

[B12] National Instutites for Health and Clinical Excellence. http://www.nice.org.uk/pdf/GDM_Allchapters_0305.pdf.

[B13] Kelson MC (2005). The NICE Patient Involvement Unit. Evidence-Based Healthcare & Public Health.

[B14] Owens DK, Nease RF (1993). Development of outcome-based practice guidelines: a method for structuring problems and synthesizing evidence. Jt Comm J Qual Improv.

[B15] Bravata DM, McDonald KM, Shojania KG, Sundaram V, Owens DK (2005). Challenges in Systematic Reviews: Synthesis of Topics Related to the Delivery, Organization, and Financing of Health Care. Ann Intern Med.

[B16] Harden A, Garcia J, Oliver S, Rees R, Shepherd J, Brunton G, Oakley A (2004). Applying systematic review methods to studies of people's views: an example from public health research. J Epidemiol Community Health.

[B17] Watts T, Merrell J, Murphy F, Williams A (2004). Breast health information needs of women from minority ethnic groups. J Adv Nurs.

[B18] (2003). AGREE Collaboration (www.agreetrust.org). Development and validation of an international appraisal instrument for assessing the quality of clinical practice guidelines: the AGREE project. Qual Saf Health Care.

[B19] Atkins D, Best D, Briss PA, Eccles M, Falck-Ytter Y, Flottorp S, Guyatt GH, Harbour RT, Haugh MC, Henry D, Hill S, Jaeschke R, Leng G, Liberati A, Magrini N, Mason J, Middleton P, Mrukowicz J, O'Connell D, Oxman AD, Phillips B, Schunemann HJ, Edejer TT, Varonen H, Vist GE, Williams JW, Zaza S (2004). Grading quality of evidence and strength of recommendations. Bmj.

[B20] Klessig J (1992). The effect of values and culture on life-support decisions. West J Med.

[B21] Blackhall LJ, Murphy ST, Frank G, Michel V, Azen S (1995). Ethnicity and attitudes toward patient autonomy. Jama.

[B22] Ruhnke GW, Wilson SR, Akamatsu T, Kinoue T, Takashima Y, Goldstein MK, Koenig BA, Hornberger JC, Raffin TA (2000). Ethical decision making and patient autonomy: a comparison of physicians and patients in Japan and the United States. Chest.

[B23] Weber E (2000). Culture and Individual Judgment and Decision Making. Applied Psychology An International Review.

[B24] Hunt LM, de Voogd KB, Castaneda H (2005). The routine and the traumatic in prenatal genetic diagnosis: does clinical information inform patient decision-making?. Patient Educ Couns.

[B25] Fischer RL, Schaeffer K, Hunter RL (2005). Attitudes of obstetrics and gynecology residents toward abortion participation: a Philadelphia area survey. Contraception.

[B26] Montori VM, Bryant SC, O'Connor AM, Jorgensen NW, Walsh EE, Smith SA (2003). Decisional attributes of patients with diabetes: the aspirin choice. Diabetes Care.

[B27] Duffy SA, Jackson FC, Schim SM, Ronis DL, Fowler KE (2006). Racial/ethnic preferences, sex preferences, and perceived discrimination related to end-of-life care. J Am Geriatr Soc.

[B28] Suurmond J, Seeleman C (2006). Shared decision-making in an intercultural context. Barriers in the interaction between physicians and immigrant patients. Patient Educ Couns.

[B29] Rebagliato M, Cuttini M, Broggin L, Berbik I, de Vonderweid U, Hansen G, Kaminski M, Kollee LA, Kucinskas A, Lenoir S, Levin A, Persson J, Reid M, Saracci R (2000). Neonatal end-of-life decision making: Physicians' attitudes and relationship with self-reported practices in 10 European countries. Jama.

[B30] Verma G, Upshur RE, Rea E, Benatar SR (2004). Critical reflections on evidence, ethics and effectiveness in the management of tuberculosis: public health and global perspectives. BMC Med Ethics.

